# Strain analysis for the prediction of the preferential nucleation sites of stacked quantum dots by combination of FEM and APT

**DOI:** 10.1186/1556-276X-8-513

**Published:** 2013-12-05

**Authors:** Jesús Hernández-Saz, Miriam Herrera, Sébastien Duguay, Sergio I Molina

**Affiliations:** 1 INNANOMAT Group, Departamento de Ciencia de los Materiales e I.M. y Q.I., Facultad de Ciencias, Universidad de Cádiz, Campus Río San Pedro, s/n, Puerto Real, Cádiz 11510, Spain; 2 GPM, Université et INSA de ROUEN, UMR CNRS 6634, BP 12, Avenue de l’université, Saint Etienne du Rouvray 76801, France

**Keywords:** Atom probe tomography, Finite elements method, Quantum dots, Indium arsenide, Self-assembled, Epitaxial growth, 81.07.Ta, 02.70.Dh, 68.37.Vj

## Abstract

The finite elements method (FEM) is a useful tool for the analysis of the strain state of semiconductor heterostructures. It has been used for the prediction of the nucleation sites of stacked quantum dots (QDs), but often using either simulated data of the atom positions or two-dimensional experimental data, in such a way that it is difficult to assess the validity of the predictions. In this work, we assess the validity of the FEM method for the prediction of stacked QD nucleation sites using three-dimensional experimental data obtained by atom probe tomography (APT). This also allows us to compare the simulation results with the one obtained experimentally. Our analysis demonstrates that FEM and APT constitute a good combination to resolve strain–stress problems of epitaxial semiconductor structures.

## Background

In the last decades, semiconductor quantum dots (QDs) have been extensively investigated because they are attractive structures for electronic and optoelectronic advanced devices [1-3]. The characteristics of these QDs can be modified by controlling the growth parameters in order to fulfil the requirements of each device. Often, well-ordered and similar-sized QDs are required in order to take advantage of their discrete energy levels for intermediate band solar cells [4], lasers [5], and photodetectors [6]. This order can be achieved by stacking several layers of QDs forming a QD matrix or superlattice. During the epitaxial growth, the strain fields of the buried QDs have a large influence in the formation of the subsequent layer as it determines the nucleation sites of the incoming stacked QDs [7,8]. The complex strain fields around a QD can produce vertical or inclined alignments [9,10], anti-alignments [11], or random distributions of the QDs [12], having a strong effect on the optoelectronic behaviour [13].

The simulation of the strain–stress fields in a semiconductor material in order to predict the location of stacked QDs lead to a better understanding of the behaviour of these complex nanostructures. The finite elements method (FEM) is a widespread tool to calculate the strain and stress fields in semiconductor nanostructures, and it has been used in the study of QDs [11,14,15], QRings [16], or QWires [17]. In order to obtain reliable predictions by FEM, the simulations should be based in experimental composition data, because of the large impact of the concentration profile of the QD systems in the strain of the structure [18]. However, because of the difficulties in obtaining three-dimensional (3D) composition data with atomic resolution, many authors use theoretical compositions [11,19], or two-dimensional (2D) experimental composition data (obtained by electron energy loss spectroscopy [20] or extrapolating composition concentration profiles measured by the lattice fringe analysis technique [21]). This makes a direct correlation between the predictions and the experimental results unfeasible, and prevents from verifying the accuracy of FEM in predicting the nucleation sites of QDs. To solve this, 3D composition data with atomic resolution should be collected. One of the most powerful techniques to obtain 3D composition data is atom probe tomography (APT). APT is an analytical technique that has the unique ability to identify and map out the positions of individual atoms from a nanostructure with an almost 3D atomic resolution, allowing the analysis of different semiconductor structures [22,23], such as QDs [24] and QRings [25].

In this paper, we have performed a strain analysis using FEM based on APT experimental data of a sample of InAs-stacked QDs. We have used the 3D compositional data obtained by APT from a layer of QDs to predict the nucleation site of the next layer of QDs, and we have compared the predictions obtained by FEM with the experimental observations by APT. Our results show that the combination of FEM with APT constitutes a powerful methodology for the analysis of the nucleation sites in stacked semiconductor QDs.

## Methods

The sample used to exemplify the study consists of InAs/GaAs-stacked QDs covered by a 2-nm In_0.2_Al_0.2_Ga_0.6_As layer grown by molecular beam epitaxy. A specimen with the needle-shaped geometry required for APT has been milled using a dual-beam FEI Quanta200 3D focused ion beam (FIB) instrument (FEI Company, Eindhoven, Netherlands) equipped with an *in situ* OMNIPROBE micromanipulator (Dallas, TX, USA), and following the procedure described in Hernández-Saz et al*.*[26]. The needle has been milled in such a way that the needle axis coincides with the [001] direction in the sample (the growth direction). In order to obtain a sharp nanometric tip (radius of about 50 nm), a sample cleaning process has been carried out with a Nvision 40 Zeiss FIB instrument (Oberkochen, Germany) using a Ga beam at 2 kV, which also reduces implantation damages. The atomic scale characterization by APT has been performed using a CAMECA LAWATAP instrument (Gennevilliers Cedex, France). About the FEM analysis, the 3D model has been defined, taking into account the composition of the structure obtained by APT using the structural mechanics module of the COMSOL software. To include the atom concentrations in the software, a discrete function of the three space variables was added. This function contains the value of the atomic concentrations of every 3 Å in the region of interest. To ensure the continuity of the data, a linear interpolation between the nearest data points is used. In order to have a negligible influence of the domain boundaries on the strain close to the QD, the Barettin et al*.*[27] criteria were followed. For this, we have considered the APT data corresponding to the lower QD layer and the barrier layer above it, and we have added simulated data around it in the growth plane and below it, in order to obtain a larger model to increase the distance from the QD to the boundaries of the model. Thus, the total simulated volume has a size of 120 × 120 × 45.5 nm, where the APT data is located in the centre, having a cylinder shape (because of the needle-shaped specimen) with a diameter of 46 nm and a height of 25 nm. The distribution of the domains in the model has been made based on the mesh density and kind of composition (experimental or simulated). For example, the base of the model consists of a subdomain with simulated data with a coarse mesh; the WL and QD are an entire subdomain with a very fine mesh; and the three last nanometers close to the surface form a subdomain of simulated data with a fine mesh. The mesh generator is based on the Delaunay algorithm, and the mesh has been designed to have higher density in the volume of the APT data and in the surface of the full domain because these are the regions of interest. Anisotropic linear elastic behaviour has been considered. Vegard's law has been assumed for the determination of the In_*x*_Al_*y*_Ga_1-*x*-*y*_As elastic constants and the lattice parameters; it is based on the atomic concentration obtained from the APT data (consequently we only import the In and Al distribution from the APT data, considering all the rest is GaAs). Initial strain was assumed to be *ϵ*_0_ = (*a*_In*x*Al*y*Ga1-*x*-*y*As_ - *a*_GaAs_)/*a*_GaAs_ in all subdomains except in the base, where *a*_*i*_ denotes the lattice parameter of *i*. The elastic properties have been taken from [28]. The elastic strain energy density (SED) can be expressed as SED = *σ*_*ij*_*ϵ*_*ij*_/2, where *σ*_*ij*_ (ϵ_*ij*_) with *i*,*j* = *x*,*y*,*z* are the components of the stress (strain) matrix (the Einstein summation convention is assumed). The normalized SED is expressed as SED/SED_max_, where SED_max_ is the maximum value of SED at the top layer surface.

## Results and discussion

**Figure 1:**
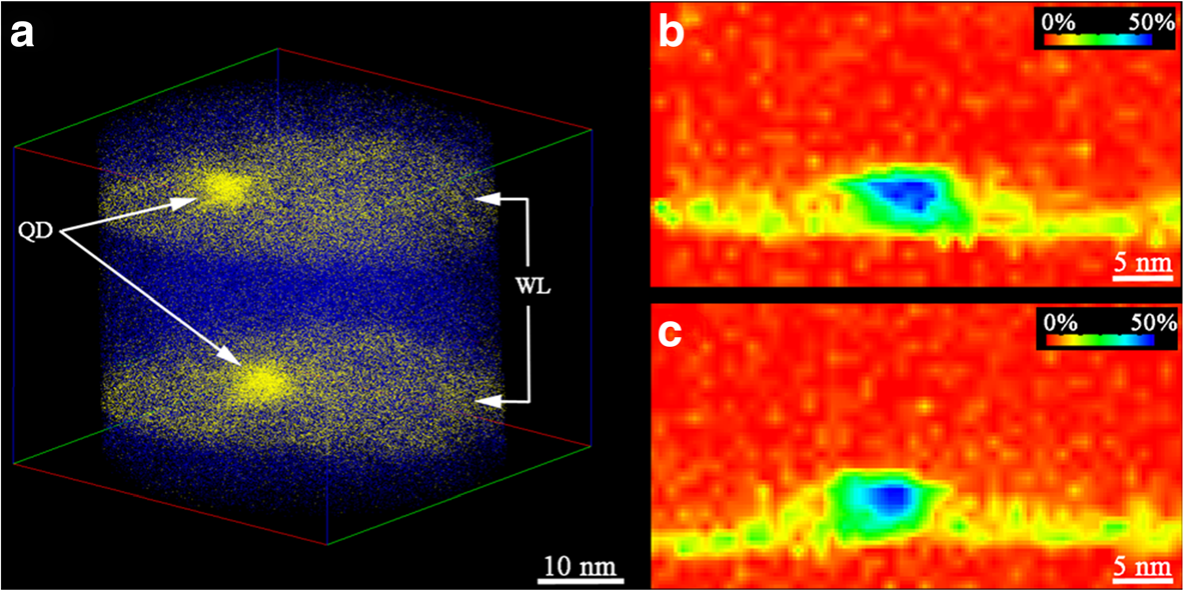
**APT data of two stacked QDs. (a)** APT data obtained from the analysed sample. In atoms are shown as yellow dots and Ga atoms as blue dots. **(b,c)** Perpendicular In composition slices of the APT data corresponding to the lower QD layer where the In inhomogeneous distribution is showed.

Figure 1a shows the APT data obtained from the fabricated needle of the sample. In atoms are shown as yellow dots and Ga atoms as blue dots (for a better visualization, only 20% of Ga atoms have been included, and none of the Al and As atoms). Our results show that the QDs (marked with arrows in the figure) are slightly asymmetric, with diameters of 9.5 ± 0.9 nm and heights of 5.6 ± 0.2 nm. Also, it should be highlighted that the APT data evidences that the QD in the second layer do not follow a vertical alignment with regard to the QD in the first layer. There is a misalignment of approximately 13° from the growth direction. Thus, our objective is to verify whether a strain analysis using FEM based on the APT data from the lower QD layer is able to predict this misalignment.

Figure 1b,c shows two perpendicular In composition slices of the APT data corresponding to the lower QD layer. The APT data in this region is the input data for the FEM analysis that will be performed next. As it can be observed in the figure, both images show an inhomogeneous In distribution, where the dark blue area indicates the higher In concentration, corresponding to the core of the QD. The absence of a uniform composition gradient from the centre of the QD in different directions prevents from the accurate theoretical simulation of the QD composition required to perform a FEM simulation that approaches the real situation. This proves that atomic scale experimental data such as those obtained from APT are essential in order to obtain realistic predictions of the QD nucleation sites from FEM analysis that can be used in the design of QD heterostructures for advanced devices.

In order to predict the nucleation site of the QD in the second layer, the chemical potential of the material during growth should be considered. In this case, the chemical potential has two major contributions: the one related to the surface energy and the one corresponding to the elastic strain. With regard to the first one, a previous analysis of the structure by transmission electron microscopy has shown that the structure grows with a flat surface, as no undulations have been observed in the wetting layers or in the surface of the structure. Because of this, the surface energy is not expected to have a major effect in the chemical potential of the structure in the prediction of the nucleation sites because prior to the formation of the second layer of QDs, the wetting layer is flat, therefore this term is neglected. As a result, the elastic strain is expected to be the determining factor for the growth process. This parameter will be calculated in this work using FEM based in the APT data.

**Figure 2:**
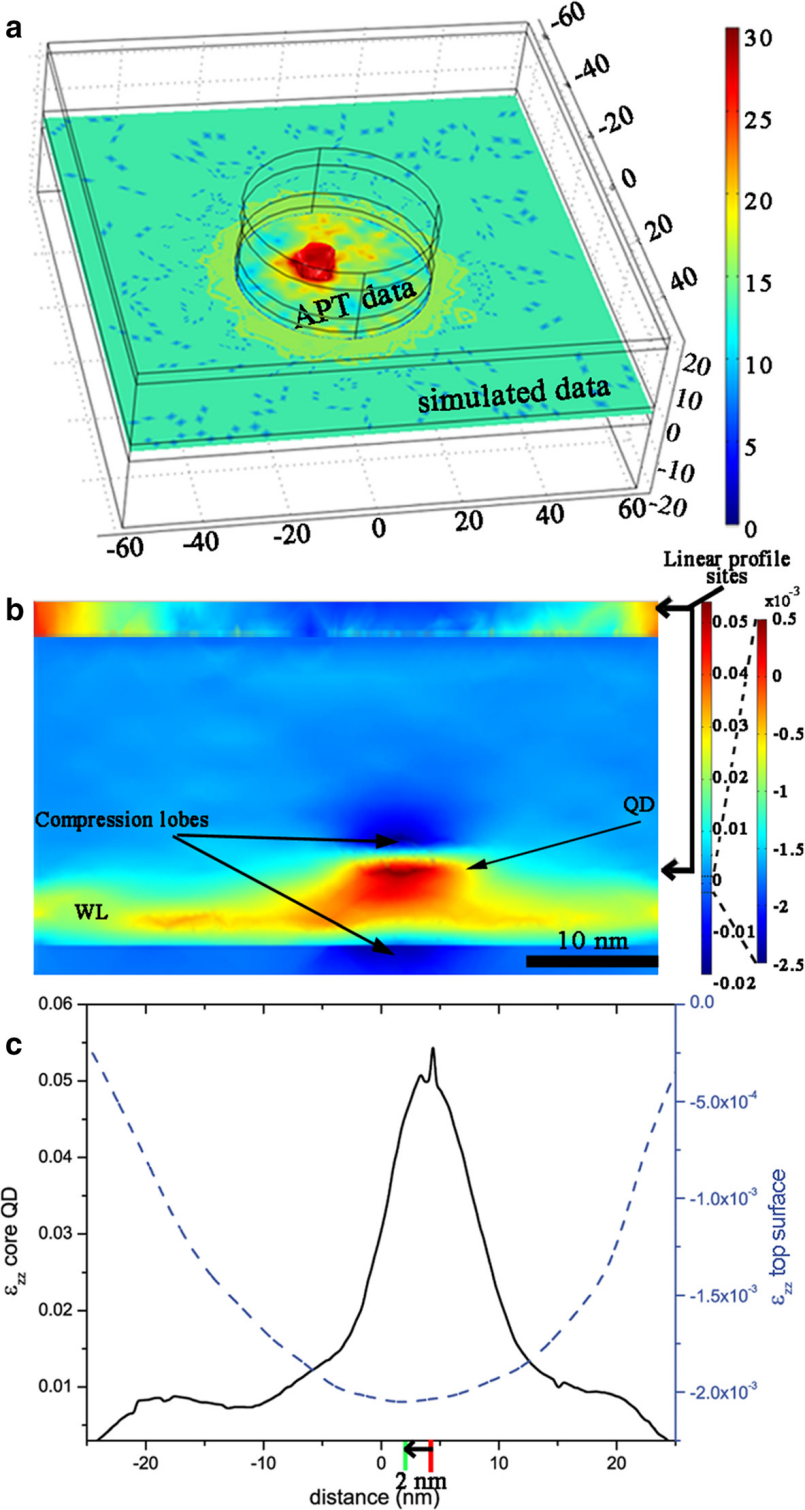
**FEM simulation with APT and simulated data of the lower QD. (a)** Slice of the input data used in the FEM simulation included in the full domain considered (in nm), where isosurfaces of 30% In are shown in red (colour scale goes from 0% In to 30% In), **(b) ***ϵ*_zz_ calculated by FEM corresponding to the area of the APT data in the model of **(a)**, and **(c)** strain line profiles along the surface of the barrier layer and along the lower QD (the green/red line marks the position of the minimum/maximum of the *ϵ*_zz_ profile).

Figure 2a shows a slice of the input data, and the domain sizes used in the FEM simulation, where the isosurfaces corresponding to a composition of 30% In in the APT data have been drawn in red colour in order to better visualize the QD. In this schematic, the limits between the APT data (corresponding to a cylindrical area because of the needle-shaped specimen, as mentioned earlier) and the simulated data added to avoid any boundary effects is highlighted. Figure 2b shows the strain in the growth direction (ϵ_zz_) calculated by FEM corresponding to the area of the APT data in the model of Figure 2a. As it can be observed, the strain due to the QD as well as the wetting layer is clearly visualized. It is worth noting that above and below the QD, two compression lobes are visible. The compression of the lattice in the growth direction in those areas is due to the expansion of the lattice in the growth plane, caused by the higher size of In atoms in comparison to Ga atoms. As it can be observed, the growth of a QD affects the GaAs area located right below the QD. Because of this, we have eliminated the 3 nm of APT data corresponding to the barrier layer right below the upper QD and we have substituted them with simulated data, to avoid any possible artefacts in our calculations. In order to predict the nucleation site of the second QD, the strain in the surface of the barrier layer needs to be analysed. However, with the scale used for visualizing the strain in the QD, the strain in that area cannot be distinguished. Because of this, we have included an inset in Figure 2b in the surface of the barrier layer also showing *ϵ*_zz_ but with a different scale in order to appreciate variations in strain. As it can be observed, an area of the material strained in compression appears in the region above the QD, which means that the lattice is expanded in the growth plane. Because of the higher size of In atoms, they will be attached preferably to these areas with higher lattice parameter; therefore, it is expected that the next QD will grow in this position. In Figure 2c, a strain line profile along the surface of the barrier layer is shown in order to assess the strain minima in that area. In this figure, a strain profile along the lower QD has also been included. As it can be observed, the strain minima in the barrier layer do not appear right above the lower QD, but there is some deviation, around 2 nm from the centre of the QD in this projection. Some deviation from the vertical alignment with the lower QD was also found in the experimental APT data. However, in order to compare the deviations found in both cases, it is necessary to analyse the situation in the growth plane.

**Figure 3:**
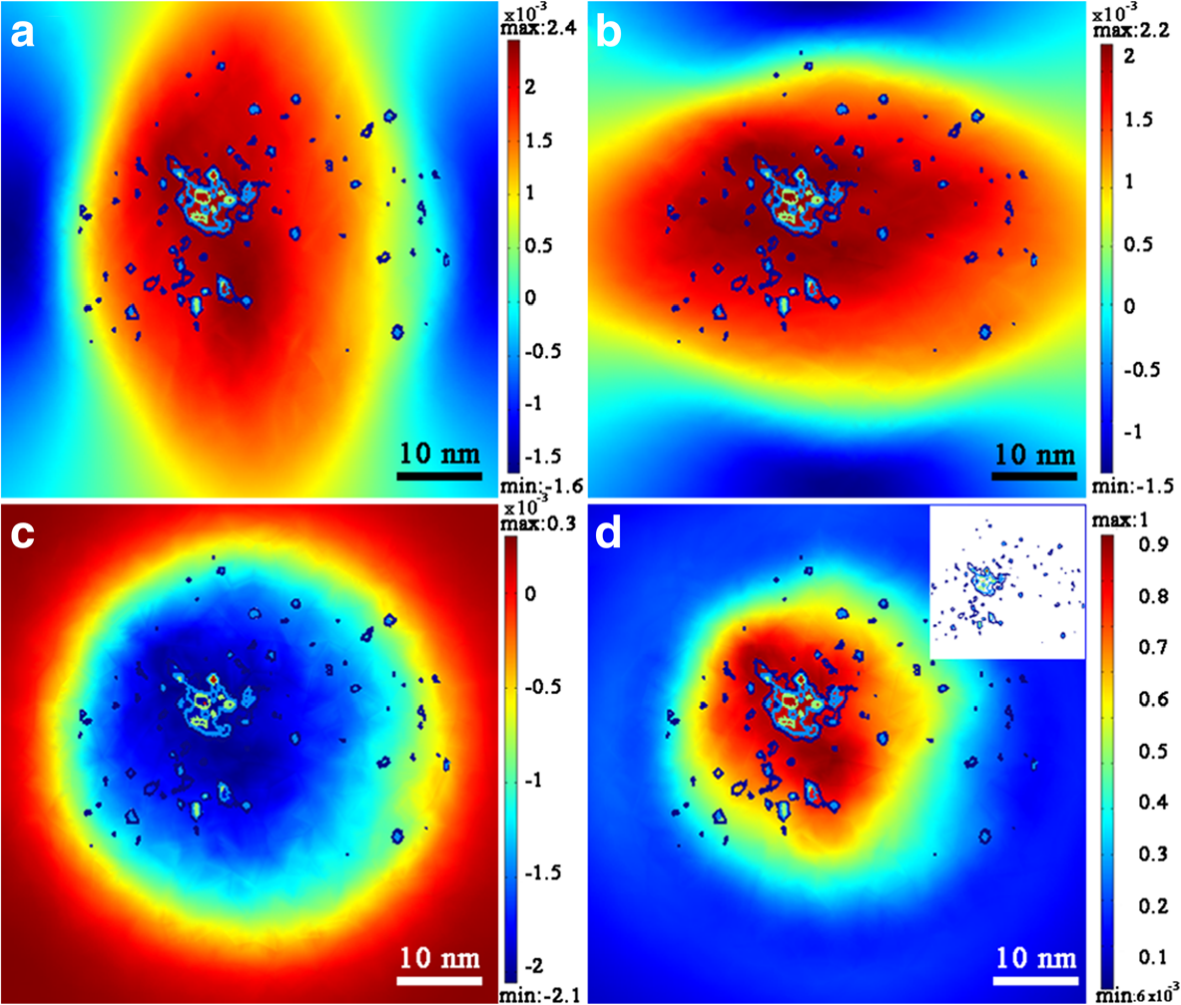
**Strain and SED maps in the growth plane of the upper QD. (a) ***ϵ*_xx_, **(b) ***ϵ*_yy_, **(c) ***ϵ*_zz_ and **(d)** normalized SED calculated in the surface of the barrier layer. Superimposed to each map, we have included the APT data corresponding to the upper layer of QDs in the form of In concentration isolines, ranging from 25% In (dark blue) to 45% In (red), in steps of 5%. In **(d)**, we have included an inset showing a complete map of the APT data for clarity.

Figure 3 shows 2D views of the strain maps calculated in the growth plane, at the surface of the barrier layer: (a) and (b) shows the strain in *x* and *y* directions (*ϵ*_xx_ and *ϵ*_yy_), which are two perpendicular axes contained in the growth plane, (c) shows *ϵ*_zz_, and (d) shows the normalized SED. In order to compare the predictions calculated by FEM with the experimental results obtained by APT, superimposed to these strain maps, we have included the APT data corresponding to the upper layer of QDs in the form of In concentration isolines, ranging from 25% In (dark blue) to 45% In (red), in steps of 5%. Also, in (d), we have included an inset showing a complete map of the APT data for clarity. As it can be observed in Figure 3a,b,c, there is a relatively wide area of similar strain where the QD would be favoured to grow, and the real QD is actually included in this area according to the APT data. Figure 3d shows the distribution of the normalized SED, which represents a compendium of strain–stress in all directions *ij* as explained earlier, and which maximum value determines the most favoured localization of the QD [29]. In this map, the area favoured for the growth of the QD has a reduced size, but the actual QD is still included in this area according to the APT experimental data [14,19]. This result shows that FEM using APT experimental data is an accurate tool for the prediction of stacked QD nucleation sites for structures where the strain component has a major effect in the chemical potential during growth. It should be mentioned that the eventual nucleation of the quantum dot is governed by a flux that drives surface adatoms from locations of higher to lower potential and the strain energy density critical value (minimum or maximum depending on the sign convention of SED [30]) is therefore the preferential site for nucleation. A more refined model would include additional parameters that typically affect the growth process, such as the surface energy [31] or kinetic effects [32]. These parameters are essential in the prediction of the nucleation sites of some semiconductor systems. For example, in InAs QWires, it has been reported that the stacking pattern is determined by the combined effect of strain and surface morphology on the growth front of the spacer layers [33]. In the structure considered in the present work, our results have shown that a simplified approximation of the chemical potential considering only the strain component is valid for obtaining accurate results.

On the other hand, our results have shown that the upper QD does not grow vertically aligned with the lower QD, but there is some deviation. Previous theoretical analyses have shown that this misalignment is, in part, related to the elastic anisotropy in the material [14], where the increase in the degree of anisotropy favours the anti-correlated island growth [19]. It has also been reported that the QD base size and density have a strong influence on this misalignment [11], although the QD shape (truncated-pyramidal or lens-shaped) may not have a major effect in the strain at the surface of the capping layer [14]. These theoretical analyses are very useful for understanding the parameters that influence the QD nucleation sites. However, they have been developed considering ideal structures, for example including perfectly symmetric QDs. Our results have shown that real QDs are far from symmetric, and small composition variations can change the strain distribution of the structure. It has been found that the strain in semiconductor structures such as QRings has a significant importance in its optoelectronic characteristics [16]. This shows that in order to understand the functional properties of real semiconductor nanostructures, it is indispensable considering real compositional data for the FEM calculations, as the APT experimental data considered in the present work.

## Conclusions

In conclusion, we have evaluated the accuracy of strain calculations by FEM using 3D atomic scale data obtained by APT for the prediction of the preferential nucleation sites of InAs stacked QDs. Our results by FEM have shown a very good agreement with our experimental observations, showing that this is a very useful tool for the analysis of the strain distribution in semiconductor systems. The combination of APT with FEM opens up the possibility of understanding the behaviour of complex semiconductor systems where strain plays a major role.

## Abbreviations

APT: Atom probe tomography; FEM: Finite elements method; FIB: Focused ion beam; GaAs: Gallium arsenide; InAs: Indium arsenide; QDs: Quantum dots; SED: Strain energy density.

## Competing interests

The authors declare that they have no competing interests.

## Authors' contributions

JHS has participated in the design of the study, prepared the experimental specimens and carried out the APT analysis with SD, performed the FEM study, taken part in discussions and in the interpretation of the results, and written the manuscript. MH has participated in the FEM data analysis; she has supervised the research and revised the manuscript, and has taken part in discussions and in the interpretation of the results. SD has taken part in discussions and in the interpretation of the results. SIM has conceived the study; he has coordinated the work and the collaboration between groups, and he has participated in its design and supervised the manuscript. All the authors have read and approved the final manuscript.

## Authors' information

JHS is a PhD student at the Universidad de Cádiz. MH is an Associate Professor at the Departamento de Ciencia de los Materiales e Ingeniería Metalúrgica y Química Inorgánica, Universidad de Cádiz. SD holds an Associate Professor at Université et INSA de ROUEN and he is the responsible of the Matériaux de la Microélectronique et de la Photonique (ER2MP) group. SIM is a full professor at the Departamento de Ciencia de los Materiales e Ingeniería Metalúrgica y Química Inorgánica, Universidad de Cádiz and the head of the Materials and Nanotechnology for Innovation group (INNANOMAT). This group belongs to the Institute of Electron Microscopy and Materials (interim stage) of the University of Cádiz.
